# Analysis of the correlation between vaginal microbiota and high-risk human papillomavirus infection and cervical lesions

**DOI:** 10.1371/journal.pone.0343027

**Published:** 2026-02-27

**Authors:** Yan Chen, Wei Qiu, Guolong Xiao, Rujing Zhang, Linlin Zhang

**Affiliations:** 1 Department of Clinical Laboratory, The Affiliated Hospital of Putian University, Putian, Fujian, China; 2 Department of Clinical Laboratory, The First Hospital of Putian City, Putian, Fujian, China; Istituto Nazionale Tumori IRCCS Fondazione Pascale, ITALY

## Abstract

The aim of this study was to evaluate changes in the vaginal microbiota and biomarkers among high-risk human papillomavirus (hrHPV)-positive women, those with hrHPV accompanied by mucositis, and patients with cervical intraepithelial neoplasia (CIN) and to establish a novel predictive model. Vaginal samples from 102 women were categorized into four groups: control group (n = 26), hrHPV-positive group (n = 22), hrHPV-positive with mucositis group (n = 26), and CIN group (n = 28). Microbiota analysis was performed using the PacBio platform with full-length 16S rDNA gene sequencing. The vaginal microbiota in the hrHPV-positive, hrHPV-positive with mucositis, and CIN groups showed significant differences compared with the healthy control group. The microbial richness in the hrHPV-positive group was significantly different from both the CIN group and healthy controls. Compared with the control group, the hrHPV-positive group exhibited significantly increased relative abundances of *Bifidobacterium*, *Escherichia-Shigella*, *Hoylesella* and nominally increased abundances of *Gardnerella*, *Prevotella*, along with a significant decrease in *Lactobacillus*. No statistically significant differences were retained between the hrHPV-positive group and the hrHPV-positive with mucositis group after FDR correction for the top 10 genera. Compared with the hrHPV-positive with mucositis group, the CIN group demonstrated significantly reduced levels of *Pseudomonas,* nominally decreased levels of *Bifidobacterium* and *Faecalibacterium*, whereas *Glutamicibacter* and *Sporosarcina* were nominally enriched. A random forest model was constructed to predict risk across groups and demonstrated good predictive performance, suggesting that vaginal microbiota may serve as valuable indicators for predicting cervical lesion risk. During hrHPV infection, significant alterations occur in the vaginal microecology, primarily characterized by an increase in pathogenic bacteria and a reduction in beneficial bacterial populations.

## Introduction

Cervical cancer is one of the most common gynecological malignancies [1,2]. According to statistics from the World Health Organization (WHO), cervical cancer is the fourth most frequently diagnosed cancer among women worldwide [3]. In recent years, the incidence of cervical cancer and precancerous lesions in China has been increasing annually and has shown a trend toward younger age groups [4,5]. Cervical intraepithelial neoplasia (CIN) collectively refers to the precancerous conditions of invasive cervical carcinoma and is clinically characterized by a continuous pathological process ranging from cervical epithelial dysplasia to carcinoma in situ, but not yet progressing to invasive squamous cell carcinoma. Human papillomavirus (HPV), belonging to the *Papillomaviridae* family, genus *Alphapapillomavirus*, is a nonenveloped, double-stranded DNA virus. Based on their oncogenic potential in cervical epithelial cells, HPV types are classified as high-risk or low-risk. Numerous studies have confirmed that persistent infection with high-risk human papillomavirus (hrHPV) is the primary cause of cervical cancer and CIN [6,7]. Although most HPV infections can be cleared spontaneously without symptoms, a subset of individuals fail to eliminate the virus effectively, leading to persistent infection [8]. Because of its anatomical location, the cervix is continuously influenced by the vaginal microbiota, and the relationship between vaginal microbial communities and HPV infection has received increasing attention in recent years [4,9]. The vaginal microecosystem is a dynamic equilibrium system composed of the vaginal anatomical structure, microbial flora, endocrine regulation, and immune mechanisms and is dominated primarily by *Lactobacillus* species [10–12] In healthy women, *lactobacilli* help defend against pathogens by maintaining a low vaginal pH and by producing hydrogen peroxide, bacteriocins, and lactic acid. These actions enhance the viscosity of cervical mucus and play a crucial role in maintaining an acidic vaginal environment and promoting natural self-cleansing functions [13]. Emerging evidence suggests that HPV infection and disease progression may be influenced by alterations in the vaginal microbiota. Reductions in *lactobacillus* abundance, along with increases in conditionally pathogenic bacteria such as *Gardnerella vaginalis* and *Atopobium*, may facilitate HPV persistence in the host and promote the development of high-grade squamous intraepithelial lesions (HSIL), thereby contributing to carcinogenesis and tumor progression [14]. The progression from HPV infection to high-grade squamous intraepithelial lesions or even cervical cancer is a stepwise process. However, the current understanding of the composition, dynamics, and clinical relevance of the vaginal microbiota during this disease progression remains limited. Effectively preventing and treating persistent HPV infection, cervical intraepithelial neoplasia, and ultimately, cervical cancer has become a significant challenge for clinicians.

In this study, 16S rDNA-based high-throughput sequencing technology was used to analyze the vaginal microbiota of healthy women, hrHPV-infected individuals, patients with hrHPV accompanied by chronic mucosal inflammation, and those with CIN. Species abundance, alpha diversity, and significantly different microbial taxa were compared among these groups. Our aim was to identify key microbial communities involved in the progression from hrHPV infection to CIN, thereby providing a theoretical foundation for the treatment of HPV-infected patients, for the development of microbiome-targeted therapeutics, and for optimizing cervical cancer screening strategies. Additionally, the aim of this study was to offer more effective and targeted prevention and treatment strategies from a microbial ecological perspective.

## Materials and methods

### Study subjects

This study enrolled 102 women from the Chinese government’s public health initiative, the “Cervical and Breast Cancer Screening Project” (specifically targeting ages 35–64), at the Affiliated Hospital of Putian University between March 1 and August 15, 2025, inclusive. Participants were selected according to the following criteria. Inclusion criteria: (1) women aged 35–64 years from urban and rural areas of Putian; (2) no history of hysterectomy or pelvic radiotherapy; (3) history of sexual activity; (4) not pregnant. Exclusion criteria: (1) sexual intercourse, vaginal douching, or intravaginal medication within 48 h before sample collection; (2) use of antibiotics or antifungal agents within the past 30 days; (3) diagnosis of invasive cervical cancer. This study was approved by the Medical Ethics Committee of the Affiliated Hospital of Putian University (approval number: PuYiFuLun [2025104]). All procedures were conducted after written informed consent was obtained from each participant.

High-risk HPV (hrHPV) genotypes, including HPV 16/18 and other 12 high-risk (OHR) types (31, 33, 35, 39, 45, 51, 52, 56, 58, 59, 66, and 68) were detected using polymerase chain reaction (PCR) combined with flow fluorescence hybridization (reagents provided by Shanghai Tumor Genesis Life Science & Technology Co., Ltd.). Cytological examinations were performed by two pathologists who independently reviewed the slides under a microscope to establish diagnoses. The results were reported according to the 2001 Bethesda System terminology, including negative for intraepithelial lesion or malignancy (NILM); low-grade squamous intraepithelial lesion (LSIL); high-grade squamous intraepithelial lesion (HSIL); atypical squamous cells of undetermined significance (ASC-US); atypical squamous cells, cannot exclude HSIL (ASC-H); atypical glandular cells (AGC); and squamous cell carcinoma (SCC). A result of NILM was considered normal, whereas any other result (collectively categorized as ASC-US+) was considered abnormal.

Women with suspected high-grade cervical lesions, glandular abnormalities, or malignancy underwent targeted biopsy under colposcopic guidance by experienced gynecologists, with multiple tissue samples obtained from the most severely affected areas. Histopathological diagnoses were made based on the WHO classification system for cervical lesions and categorized into five groups: normal, cervical intraepithelial neoplasia grade 1 (CIN1), CIN2, CIN3, and cervical cancer.

**Fig 1:**
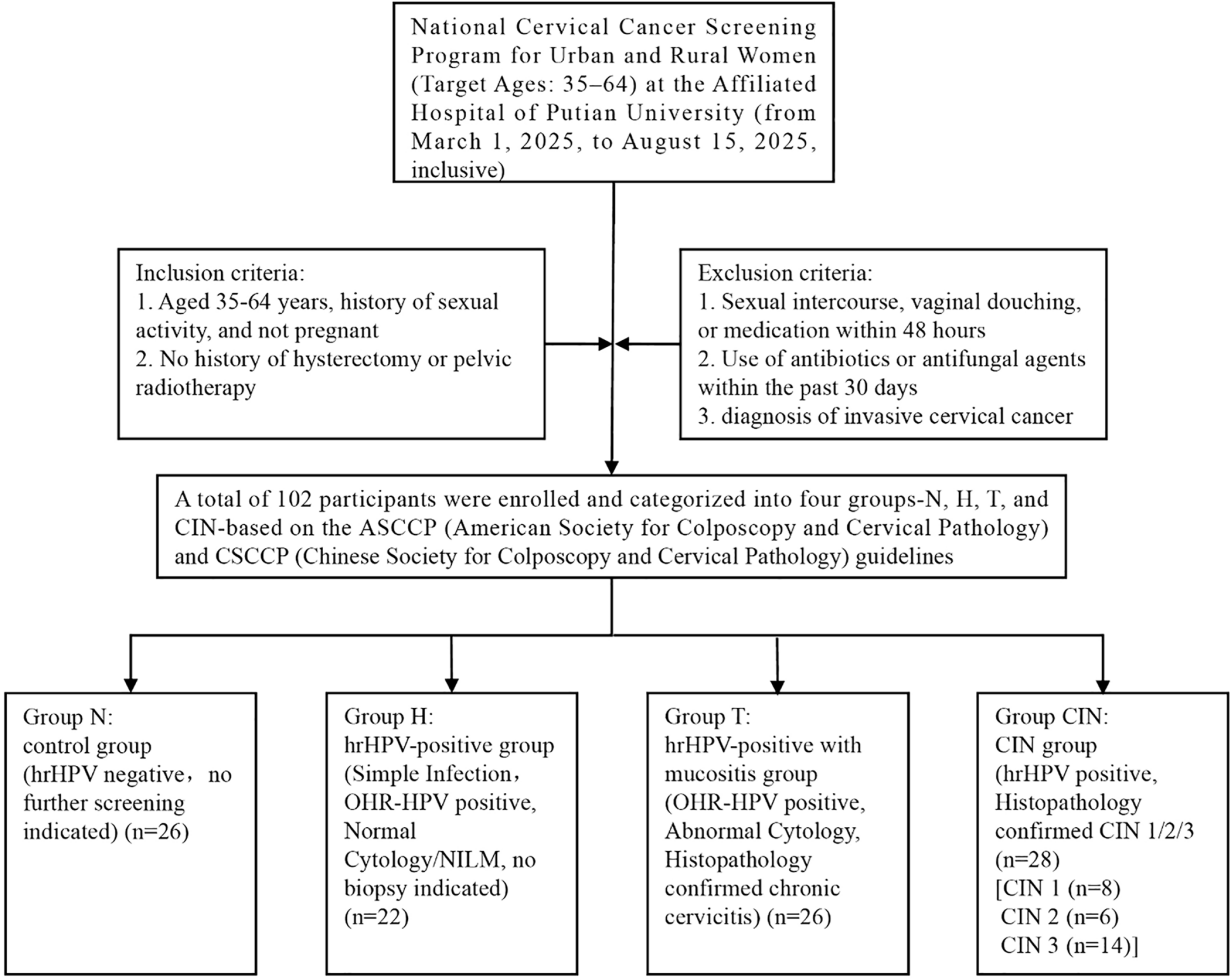
Flowchart of the study protocol.

Participants were categorized into four groups based on the ASCCP (American Society for Colposcopy and Cervical Pathology) and CSCCP (Chinese Society for Colposcopy and Cervical Pathology) guidelines. Control group (Group N): hrHPV-negative women. HRHPV-positive group (Group H) (Simple Infection): Women with OHR HPV and normal cytology (NILM) (no biopsy required). HRHPV-positive with mucositis group (Group T): Women with OHR HPV and abnormal cytology, histopathologically confirmed as chronic cervicitis. CIN group (Group CIN): hrHPV-positive women histopathologically diagnosed with CIN 1 (n = 8), CIN 2 (n = 6), and CIN 3 (n = 14)). The study flowchart is shown in Fig 1.

### Sample collection

All vaginal samples were collected at the initial examination, strictly prior to any treatment (including probiotics). Participants were positioned in the lithotomy position, and the cervix was fully exposed using a sterile speculum. A single-use sterile swab was inserted into the upper one-third of the vagina and gently rotated for 10–15 s to collect vaginal secretions and epithelial cells. The swab was then withdrawn, and the shaft was broken off into a sterile cryotube. All samples were immediately stored at −80°C until further processing.

### 16S rDNA-based amplicon sequencing

Total genomic DNA was extracted from the samples using the CTAB method. DNA concentration and purity were assessed using 1% agarose gel electrophoresis. Based on the concentration, DNA was diluted to 1 ng/µL using sterile water. The V3–V4 hypervariable regions of the 16S rRNA gene were amplified using specific primers 341F (5’-CCTAYGGGRBGCASCAG-3’) and 806R (5’-GGACTACNNGGGTATCTAAT-3’), each containing a unique barcode. An equal volume of 1 × TAE buffer was mixed with the PCR products, which were subjected to electrophoresis on 2% agarose gel to confirm amplification. PCR products were pooled in equimolar ratios and purified using the Universal DNA Purification Kit (TianGen, China). Sequencing libraries were prepared using the NEBNext® Ultra^TM^ DNA Library Prep Kit (Illumina, USA) following the manufacturer’s protocol, and index codes were added. Library quality was assessed on the Agilent 5400 system (Agilent Technologies Co. Ltd., USA). Finally, sequencing was performed on an Illumina platform, generating 250 bp paired-end reads.

### Bioinformatics analysis

Raw paired-end sequencing reads were first quality-filtered using *fastp* (v0.19.6) and then merged using *FLASH* (v1.2.11). After quality control and merging, sequencing denoising was performed using the DADA2 plugin in the QIIME2 pipeline with default parameters. All contaminant sequences related to chloroplasts and mitochondria were removed. To minimize the influence of sequencing depth on alpha and beta diversity analyses, sequence counts across all samples were rarefied to 20,000 reads per sample. After rarefaction, the average sequence coverage for each sample remained high (99.09%). Taxonomic classification of amplicon sequence variants (ASVs) was conducted using the Naive Bayes classifier in QIIME2, based on the SILVA 16S rRNA gene reference database (v138.2). Functional predictions of microbial communities were performed using PICRUSt2 (v2.2.0).

### Statistical analysis

Taxonomic abundances at the phylum, class, order, family, genus, and species levels were compared among the four groups using the Kruskal-Wallis test, while pairwise comparisons between two groups were performed using the Wilcoxon rank-sum test (R stats package). After controlling for the false discovery rate (FDR), adjusted *p-value* (*P*_*adj*_) of less than 0.05 was viewed as statistically significant. Unadjusted *p-value* (*p* < 0.05) was considered nominally significant. Additionally, 95% confidence intervals (CI) were calculated using the bootstrap method. Alpha diversity indices, including Chao1 and ACE, were calculated using mothur® software (v1.30.2). Intergroup differences in alpha diversity were assessed using the Kruskal-Wallis test, followed by post-hoc pairwise comparisons. Principal coordinates analysis (PCoA) based on Bray-Curtis dissimilarity distances was performed to evaluate similarities of microbial community structure among samples. Permutational multivariate analysis of variance (PERMANOVA) was employed as a nonparametric test to determine whether the differences in microbial community composition between groups were statistically significant.

## Results

### Comparison of demographic characteristics among groups

Characteristics of 102 participants were analyzed. There were no statistical differences in general clinical data among the groups including Age, Menopausal history, Gravidity, Cleanliness and *Gardnerella vaginalis* (Table 1).

**Table 1:** Comparison of demographic characteristics among groups.

characteristics	N(n = 26)	H(n = 22)	T(n = 26)	CIN(n = 28)	x2 value	P value
Age					0.3971	0.5286
≤50	19	10	17	19		
>50	7	12	9	9		
Menopausal history					0.01420	0.9051
premenopausal	20	12	18	22		
postmenopausal	6	10	8	6		
Gravidity					0.04	0.999
1	2	4	9	0		
2	10	12	10	11		
3	6	4	5	11		
4	6	1	1	2		
5	1	1	1	2		
6	1	0	0	2		
Cleanliness					0.01548	0.9010
Normal	12	15	20	19		
Abnormal	14	7	6	9		
*Gardnerella vaginalis*					0.01071	0.9176
+	3	3	0	0		
–	23	19	26	28		

Vaginal cleanliness: Normal, Grades I–II (Lactobacillus-dominant, few leukocytes); Abnormal, Grades III–IV (reduced Lactobacillus, abundant leukocytes).

### Sequencing depth analysis

Rarefaction curves based on the Sobs index showed that the curves of all samples reached a stable plateau with increasing sequencing depth, indicating that the sequencing data volume was sufficient for subsequent analysis of community composition and diversity (S1 Fig).

### Species abundance analysis

**Fig 2:**
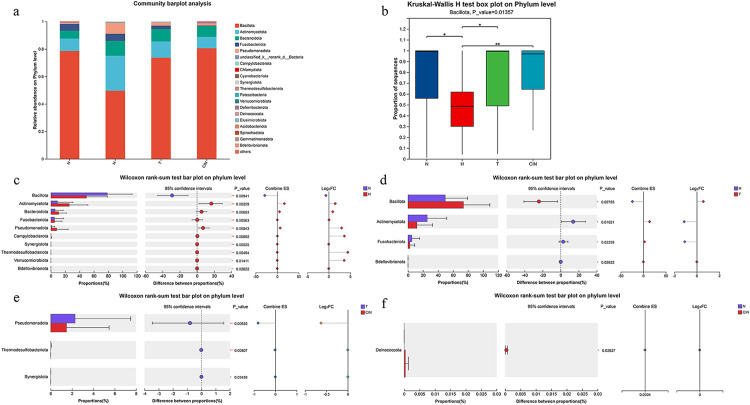
Phylum-level composition and differential analysis. (a) relative abundance of top 20 phyla in four groups. **(b)** Abundance variation of *Bacillota*. **(c-f)** Pairwise comparisons of differentially abundant phyla between groups: **(c)** N vs H, **(d)** H vs T, **(e)** T vs CIN, and **(f)** N vs CIN.

Bar plots illustrating the relative abundances of the predominant vaginal microbial taxa across the four groups at the phylum level are shown in Fig 2. *Bacillota* was the most dominant bacterial group in all groups, with median relative abundances of 99.39% (IQR: 55.92%–99.94%) in the control group, 48.53% (IQR: 30.04%–61.78%) in the hrHPV-positive group, 99.47% (IQR: 49.00%–99.88%) in the hrHPV-positive with mucositis group, and 96.98% (IQR: 64.27%–99.89%) in the CIN group (Fig 2b). However, no statistically significant differences were observed among the groups (*p =* 0.01, *P*_*adj*_ = 0.05). The 10 most abundant bacterial phyla identified in the vaginal microbiota were *Bacillota, Actinomycetota, Bacteroidota, Fusobacteriota, Pseudomonadota, Campylobacterota, Chlamydiota, Cyanobacteriota*, and *Synergistota* (Fig 2a).Wilcoxon rank-sum tests revealed that, compared to the control group, the hrHPV-positive group showed significantly higher relative abundances of *Actinomycetota* (Mean: 9.00% vs 25.54%)*, Bacteroidota* (5.69% vs 10.86%), and *Pseudomonadota* (1.00% vs 7.89%), along with a significant reduction in *Bacillota* (78.59% vs 49.56%) (all *P*_*adj*_ < 0.05) and a nominal increase in *Deinococcota* (0 vs 0.0004%) in the CIN group (*p* < 0.05, *P*_*adj*_ > 0.05). Compared with the CIN group, the hrHPV-positive with mucositis group exhibited nominally higher abundances of *Pseudomonadota* (1.49% vs 2.29%) and *Thermodesulfobacteriota* (0 vs 0.02%) (all *p* < 0.05, *P*_*adj*_ > 0.05) (Fig 2c-f and S1 Table).

**Fig 3:**
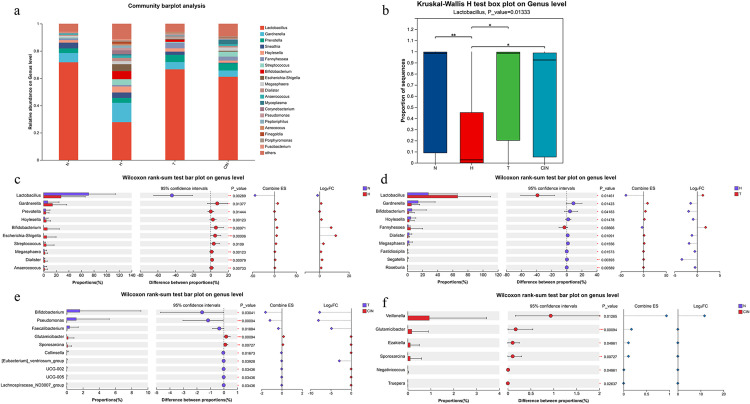
Genus-level composition and differential analysis. (a) relative abundance of top 10 genera in four groups. **(b)** Abundance variation of *Lactobacillus*. **(c-f)** Pairwise comparisons of differentially abundant genera between groups: **(c)** N vs H, **(d)** H vs T, **(e)** T vs CIN, and **(f)** N vs CIN.

At the genus level, *Lactobacillus* was the most abundant genus across all groups, with median relative abundances of 98.53% (IQR: 8.98%–99.61%) (control), 27.05% (IQR: 0.07%–45.24%) (hrHPV-positive), 98.58% (IQR: 20.19%–99.53%) (hrHPV-positive with mucositis), and 92.45% (IQR: 5.27%–98.79%) (CIN) (Fig 3b). The other 10 predominant genera included *Gardnerella, Prevotella, Sneathia, Hoylesella, Fannyhessea, Streptococcus, Bifidobacterium, Escherichia-Shigella*, and *Megasphaera* (Fig 3a). Further pairwise comparisons revealed significant differences in genus-level composition among the groups. Compared with the control group, the hrHPV-positive group showed significantly increased abundances of *Hoylesella* (Mean: 1.86% vs 4.41%)*, Bifidobacterium* (0.03% vs 9.95%), *Escherichia-Shigella* (0.002% vs 5.03%) (all *P*_*adj*_ < 0.05) and nominally increased abundances of *Gardnerella* (6.78% vs 14.21%), *Prevotella* (3.40% vs 3.57%) (all *p* < 0.05, *P*_*adj*_ > 0.05), along with a significant reduction in *Lactobacillus* (71.71% vs 27.77%, *P*_*adj*_ < 0.05). Regarding the top 10 genera, no statistically significant differences were retained between the hrHPV-positive group and the hrHPV-positive with mucositis group after FDR correction (*P*_*adj*_ > 0.05). In contrast, compared with the hrHPV-positive with mucositis group, the CIN group showed significantly reduced levels of *Pseudomonas* (1.17% vs 0.004%, *P*_*adj*_ < 0.05), nominally decreased levels of *Bifidobacterium* (1.62% vs 0.007%) and *Faecalibacterium* (0.35% vs 0.01%) (all *p* < 0.05, *P*_*adj*_ > 0.05), whereas *Glutamicibacter* (0 vs 0.17%) and *Sporosarcina* (0 vs 0.11%) were nominally enriched (all *p* < 0.05, *P*_*adj*_ > 0.05) (Fig 3c–f and S1 Table).

### Microbial diversity analysis

**Fig 4:**
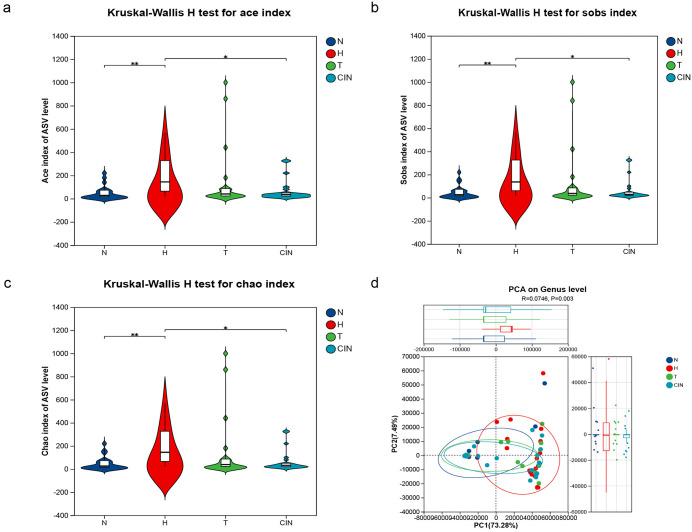
The microbial diversity analysis in different groups. **(a)** Ace index box chart for each group. **(b)** Sob index box chart for each group. **(c)** Chao1 index box chart for each group.(*P < 0.05,**P < 0.01). **(d)** The microbial Beta diversity analysis in different groups.

Kruskal-Wallis test were used to compare Chao1, ACE, and Sob indices (alpha diversity) across groups. The hrHPV-positive group exhibited significantly higher alpha diversity compared with all other groups. Data are presented as median [IQR]. Specifically, the Chao1 index in the hrHPV-positive group (145.13 [IQR: 64–364.21]) was significantly higher than in the healthy control group (26.71 [IQR: 17–73]; *p* < 0.01) and the CIN group (32.25 [IQR: 21–50.5]; *p* < 0.05) (Fig 4c). Similarly, the ACE index was significantly elevated in the hrHPV-positive group (144.42 [IQR: 62.34–364.77]) compared with both the healthy control (27.72 [IQR: 17.4–73]; *p* < 0.01) and CIN groups (34.77 [IQR: 22.03–50.36]; *p* < 0.05) (Fig 4a). No significant differences in these indices were observed among the control, hrhpv-positive with mucositis and CIN groups. Analysis of subgroups according to cervical lesion severity revealed a decreasing trend in Sob index values with increasing disease severity, reaching the lowest value in the CIN group (28.5 [IQR: 20.5–48]), with statistical significance (*p* < 0.05) (Fig 4b).

Beta diversity was assessed using principal component analysis (PCA) based on Euclidean distance. The results showed that samples from the control group clustered more tightly than those from hrHPV-positive group, indicating higher similarity in vaginal microbial composition within the control group (Fig 4d) and confirmed by statistical testing on the distance matrices Box plots representing the distribution of weighted UniFrac distances. The Kruskal-Wallis H test revealed a highly significant difference (p = 4.611e-11) (S2 Fig).

### Correlations between microbiota and clinical/demographic factors

**Fig 5:**
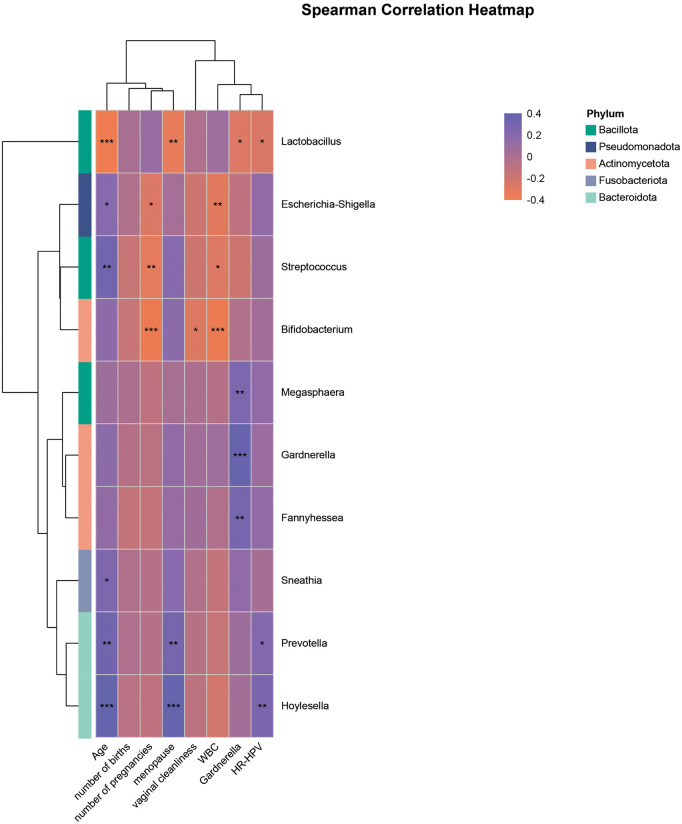
A heatmap of Spearman correlation coefficients between the top 10 genera and clinical factors. (* 0.01 < *P* ≤ 0.05,**0.001 < *P* ≤ 0.01,***P ≤ 0.001).

Spearman correlation analysis was performed across the four groups to explore associations between the vaginal microbiota and demographic characteristics, parity, gravidity, and routine vaginal discharge parameters. *Lactobacillus* abundance was negatively correlated with age (*p* = 0.001), menopausal status (*p* = 0.01), *Gardnerella* abundance (*p* = 0.05), and HR-HPV infection rate (*p* = 0.05). *Escherichia-Shigella* abundance was negatively correlated with the number of pregnancies (*p* < 0.05) and white blood cell (WBC) count (*p* < 0.01). The number of pregnancies, vaginal cleanliness grade, and WBC count were all negatively correlated with *Bifidobacterium abundance* (*p* < 0.001; p < 0.05; *p* < 0.001, respectively). *Gardnerella* showed positive correlations with *Megasphaera* and *Fannyhessea* (*p* < 0.01 for both). *Prevotella* abundance was positively associated with age (*p* < 0.01), menopause (*p* < 0.01), and HR-HPV infection (*p* < 0.05). *Hoylesella* was positively correlated with age (*p* < 0.001), *Megasphaera* (*p* < 0.001), and HR-HPV infection (*p* < 0.01) (Fig 5).

### Random forest-based risk prediction model for cervical lesions

**Fig 6:**
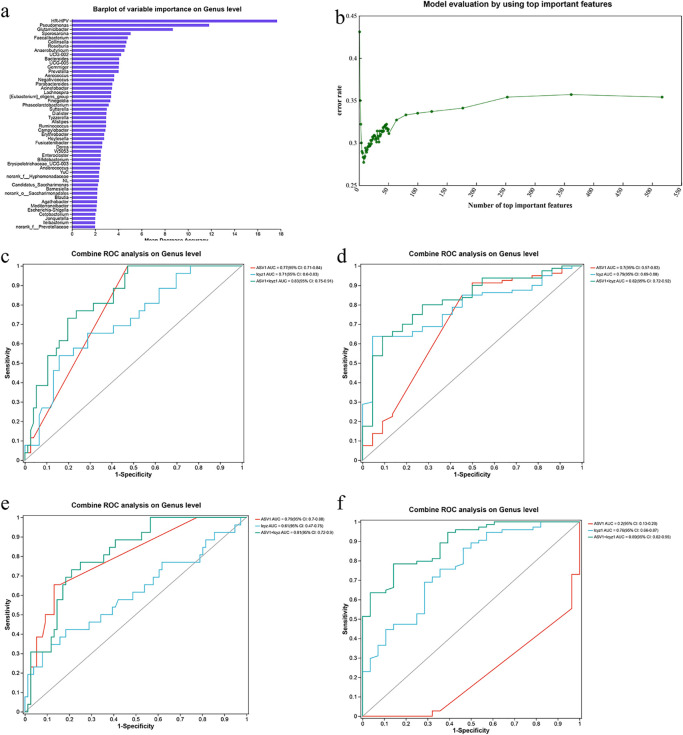
Random Forest and ROC Analysis. **(a)** Random-forest graph of top 50 different bacterial communities between each group. **(b)** Model evaluation with ten-fold cross-validation using varying numbers of important features. **(c)** ROC curve of Group **N. (d)** ROC curve of Group **H. (e)** ROC curve of Group **T. (f)** ROC curve of Group CIN.

Because cervical lesion development is influenced by multiple factors, a random forest-based risk prediction model was constructed that integrated both vaginal microbial composition and host demographic/clinical variables, including age, number of births, number of pregnancies, menopausal status, vaginal cleanliness, WBC count, *Gardnerella* abundance, and HR-HPV status. Variable importance was ranked using the random forest algorithm, and the top 50 most influential features were selected (Fig 6a). Ten-fold cross-validation was performed to determine the optimal number of variables for the final model. The cross-validation error rate progressively declined as the number of variables increased, reaching a minimum (27.7%) when eight variables were included (Fig 6b).

A final random forest model was then built using these eight selected predictors, and its performance was evaluated using ROC curve analysis (Fig 6c-f). The area under the ROC curve (AUC) was used to assess predictive accuracy. The integrated model combining both microbial and host factors demonstrated improved performance. The highest prediction accuracy was achieved for the CIN group (AUC = 0.89), followed by the hrHPV-positive group (AUC = 0.82), the hrHPV-positive with mucositis group (AUC = 0.81), and the control group (AUC = 0.83), indicating strong potential for using this integrated model in clinical risk stratification.

## Discussion

The female reproductive tract is an open channel that communicates directly with the external environment, making it susceptible to invasion by exogenous microorganisms and thereby increasing the risk of infectious diseases such as human papillomavirus (HPV) infection [15]. Persistent high-risk HPV (HR-HPV) infection is a well-established cause of cervical cancer [5]. The vaginal microbiota, as a key component of the lower genital tract microecosystem, plays a crucial role in the development and progression of cervical lesions. Vaginal microbial homeostasis is influenced by various factors, including age, menstrual cycle, medication use, vaginal douching, and contraceptive methods [16]. It represents a dynamic equilibrium maintained through complex interactions between host microbiota and environmental conditions [17]. Once this balance is disrupted and local immune defenses are compromised, pathogenic microbes may invade the reproductive tract, reducing the body’s ability to clear HPV and other pathogens. Increasing evidence suggests that alterations in the vaginal microbiome play a significant role in the pathogenesis of cervical lesions induced by HPV infection.

In recent years, research on vaginal microbial community diversity has become increasingly prominent. *Alpha* diversity is a fundamental metric used to assess microbial community richness and diversity. Diversity indices reflect community heterogeneity, whereas richness measures the number of species within a single sample. Increased vaginal microbial diversity is often considered indicative of dysbiosis and may be associated with the progression of cervical lesions [17]. However, previous studies have reported inconsistent findings regarding the relationship between vaginal microbial diversity and cervical disease [18]. One study conducted in a Chinese cohort demonstrated that increased vaginal microbial diversity was closely associated with HPV infection and the development of cervical intraepithelial neoplasia (CIN). In contrast, other studies found no significant differences in bacterial abundance or diversity across different stages of cervical lesions, although HPV infection itself was linked to increased microbial richness and diversity [19]. In our study, the Chao1 index was used to estimate microbial richness. Significantly higher microbial richness was observed in the hrHPV-positive group compared with both the CIN group and healthy controls. These discrepancies across studies may be attributable to variations in geographic regions, ethnic backgrounds, sample sizes, and sampling methodology [20]. A large-scale study encompassing diverse ethnic groups revealed that Caribbean African women had a fourfold higher risk of increased microbial diversity and vaginal dysbiosis compared with European/Caucasian and African women, highlighting ethnicity as a key determinant of vaginal microbiota composition [21]. As all participants in our study were Han Chinese, our findings reflect characteristics specific to this population. Therefore, multicenter, global studies are warranted to better understand the generalizability of these observations.

The application of high-throughput sequencing technology has greatly advanced our understanding of the vaginal microbiome, enabling more precise investigation into its relationship with cervical pathology. Our results showed that *Bacillota* was the dominant phylum and *Lactobacillus* the predominant genus in the healthy vaginal microbiota—findings consistent with previous studies [17]. Compared with the control group, the hrHPV-positive group exhibited significantly increased relative abundances of *Actinomycetota, Bacteroidota*, and *Pseudomonadota* (*P*_*adj*_ < 0.05)*,* indicating substantial alterations in microbial structure during disease states. Previous studies have also reported elevated levels of *Bacteroidota* and *Actinomycetota* along with reduced *Lactobacillu*s in HPV-infected individuals [22]. Such dysbiosis may disrupt vaginal homeostasis, increasing susceptibility to HPV infection or promoting viral persistence. Recent evidence further indicates that *Pseudomonadota* is significantly enriched in infertile women and may impair fertility by disrupting the *Lactobacillus*-dominated ecosystem [23]. Members of this phylum may interact with *Lactobacillus* and *Candida albicans* through competitive inhibition or metabolic by-products;their overgrowth has been linked to vaginal inflammation and recurrent implantation failure, among other reproductive health issues.

Designed as an exploratory study based on an hrHPV-primary sequential screening strategy, this research aims to identify risk factors for abnormal outcomes and establish a diagnostic prediction model to optimize screening workflows. However, given the high inter-individual heterogeneity characteristic of the vaginal microbiome [24] and the constraints of our sample size, strictly adhering to False Discovery Rate (FDR) correction poses a risk of discarding potentially valuable biomarkers (increasing Type II errors). Crucially, our Random Forest analysis demonstrated that *Glutamicibacter*, *Sporosarcina*, and *Faecalibacterium* ranked among the top five most influential features for disease classification, despite lacking statistical significance after FDR adjustment. This discrepancy highlights the inherent limitations of univariate statistics in capturing non-linear microbial interactions. Consequently, we retained these nominally significant taxa as candidate features–a methodological strategy supported by recent high-impact research [25].

In our study, the hrHPV-positive group showed significantly higher abundances of *Hoylesella, Bifidobacterium, Escherichia-Shigella* (*P*_*adj*_ < 0.05) and nominal increase abundances of *Gardnerella*, *Prevotella* (*p* < 0.05, *P*_*adj*_ > 0.05)*,* along with a marked decrease in *Lactobacillus* (*P*_*adj*_ < 0.05), compared with the control group. This aligns with previous reports showing *Lactobacillus* dominance in healthy vaginal microbiota. However, we also observed substantial inter-individual heterogeneity within groups (as indicated by the wide IQR). This likely reflects the natural diversity of vaginal Community State Types (CSTs) [20,24]. Furthermore, across the spectrum of hrHPV infection (ranging from latent infection and mucositis to CIN), this heterogeneity is amplified by the dynamic transition of the microbiome during disease progression or inflammatory instability (e.g., shifting from *Lactobacillus*-rich to dysbiotic states), creating a mixed population with divergent microbial profiles. Notably, compared with the hrHPV-only group, the hrHPV-positive with mucositis group exhibited no statistically significant differences (*P*_*adj*_ > 0.05) in the top 10 genera. This lack of robust variation suggests that mucositis per se may not drastically alter the core vaginal microbiome structure in hrHPV-positive women, or that any potential alterations are subtle and would require larger cohorts to detect. Compared with the hrHPV-positive with mucositis group, the CIN group showed significantly reduced levels of *Pseudomonas* (*P*_*adj*_ < 0.05), and nominally decreased levels of *Bifidobacterium* and *Faecalibacterium* (*p* < 0.05, *P*_*adj*_ > 0.05), whereas *Glutamicibacter* and *Sporosarcina* were nominally enriched (*p* < 0.05, *P*_*adj*_ > 0.05). *Gardnerella* is one of the most commonly identified microorganisms in vaginal samples from women with bacterial vaginosis (BV) and is frequently detected in HPV-infected and cervical cancer patients. It can form dense biofilms adherent to vaginal epithelial cells, release hemolysins that trigger inflammatory responses, and produce virulence factors such as sialidase (SNA) and hemolysin, which damage epithelial cells and compromise mucosal barriers—facilitating adhesion and invasion by pathogens like HPV [26]. Although the increase in *Gardnerella* showed only nominal significance (*p* < 0.05), its potential role as a key cofactor in HPV persistence and cervical carcinogenesis is well-supported by literature. *Prevotella*, an anaerobic genus, which was also identified as an informative feature in our Random Forest model, has been shown to activate NF-κB signaling and induce pro-inflammatory chemokines. It produces multiple virulence factors—including hemolysin, sialidase, proline aminopeptidase, and putrescine—that promote epithelial cell death, impair barrier integrity, and contribute to persistent HR-HPV infection and CIN development [18,27]*. Hoylesella* is a recently recognized member of the vaginal microbiota associated with HPV infection. Emerging studies indicate that *Hoylesella* is significantly more abundant in HR-HPV-positive women than in healthy controls, suggesting it may promote persistent infection by disrupting microbial balance. This bacterium often acts synergistically with *Gardnerella* and *Prevotella*, leading to depletion of *Lactobacillus* and weakening of the vagina’s natural defense mechanisms (Prognostic and diagnostic value of next-generation sequencing in female reproductive tract infection and HPV persistence). Interestingly, *Escherichia-Shigella* has been reported to exhibit potential protective effects during the CIN stage. It may indirectly modulate systemic immunity by regulating gut microbiota balance, thereby suppressing HPV-related cervical lesions. In our correlation analysis, *Escherichia-Shigella* abundance was negatively correlated with white blood cell (WBC) count, suggesting a possible anti-inflammatory role that may help limit disease progression. Currently, limited evidence directly links *Faecalibacterium* to the vaginal microbiome. This genus is primarily found in the gut, where it functions as a major beneficial bacterium with well-known anti-inflammatory properties [28]. It contributes to intestinal and systemic immune homeostasis through butyrate production and immunomodulatory effects, playing an important role in gut health and immune regulation. The observed reduction of *Faecalibacterium* in the CIN group in our study suggests a potential association between its depletion and the development of vaginal inflammatory conditions or systemic immune dysregulation in advanced cervical lesions. In our analysis of correlations between microbiota and clinical factors, increasing age, gravidity, parity, and number of abortions are associated with disruption of the vaginal microenvironment—characterized by decreased *Lactobacillus* abundance and increased proportions of anaerobic bacteria such as *Prevotella* and *Gardnerella* [29]. Severe systemic and local inflammation was closely linked to vaginal microbial imbalance, further emphasizing the interplay between host physiology, microbial ecology, and disease progression.

This study presents several notable strengths and novel contributions to the existing literature. Primarily, to our knowledge, this is the first study to integrate microbiome analysis specifically with the key nodes of the cervical cancer sequential screening strategy in the Putian cohort. By utilizing a Random Forest model, we successfully identified characteristic microbial signatures for risk prediction, extending beyond simple abundance comparisons. Furthermore, our analysis elucidated the critical interplay between host physiology (e.g., age, reproductive history) and vaginal dysbiosis, providing a more holistic view of the disease microenvironment. However, we acknowledge certain limitations. First, the restriction to women aged 35–64 years, while aligning with the national “Cervical and Breast Cancer Screening Project,” limits the generalizability of our findings to younger populations. Second, the relatively limited sample size, coupled with the inherent inter-individual heterogeneity of the vaginal microbiome (as evidenced by the wide IQR), restricted the statistical power. Consequently, several biologically relevant taxa showing nominal significance did not survive rigorous FDR correction. Third, this sample size constraint precluded granular subgroup analyses based on histological grades (CIN 1–3) or specific HPV genotypes, which may have masked subtle microbial variations. Future large-scale, multi-center longitudinal studies are warranted to validate these findings and address these gaps.

## Conclusions

In conclusion, this study reveals that vaginal microbiota changes across the spectrum of hrHPV infection and cervical lesions are characterized by complex, non-linear fluctuations rather than simple linear depletion. Our findings offer two key implications for clinical practice: first, the identified microbial signatures support the development of auxiliary biomarkers to optimize risk stratification in screening programs; second, the distinct dysbiotic profiles observed in simple hrHPV infection, mucositis and CIN provide a theoretical basis for targeted microbiota-based interventions. Future efforts should focus on validating these models in larger, longitudinal cohorts to translate these ecological insights into precision medicine strategies for cervical cancer prevention.

## Supporting information

S1 Data Underlying data for all figures and statistical analysis. (XLSX)

S1 Table List of differentially abundant taxa identified by pairwise comparisons among the four groups. (XLSX)

S1 Fig Rarefaction curves based on the Sobs index. (TIF)

S2 Fig Beta diversity difference analysis. (TIFF)
