# Supplementary figures and images for: Analysis of the correlation between vaginal microbiota and high-risk human papillomavirus infection and cervical lesions
Source: PLoS One. 2026 Feb 27;21(2):e0343027. doi: 10.1371/journal.pone.0343027 (PMC12948077; doi:10.1371/journal.pone.0343027)

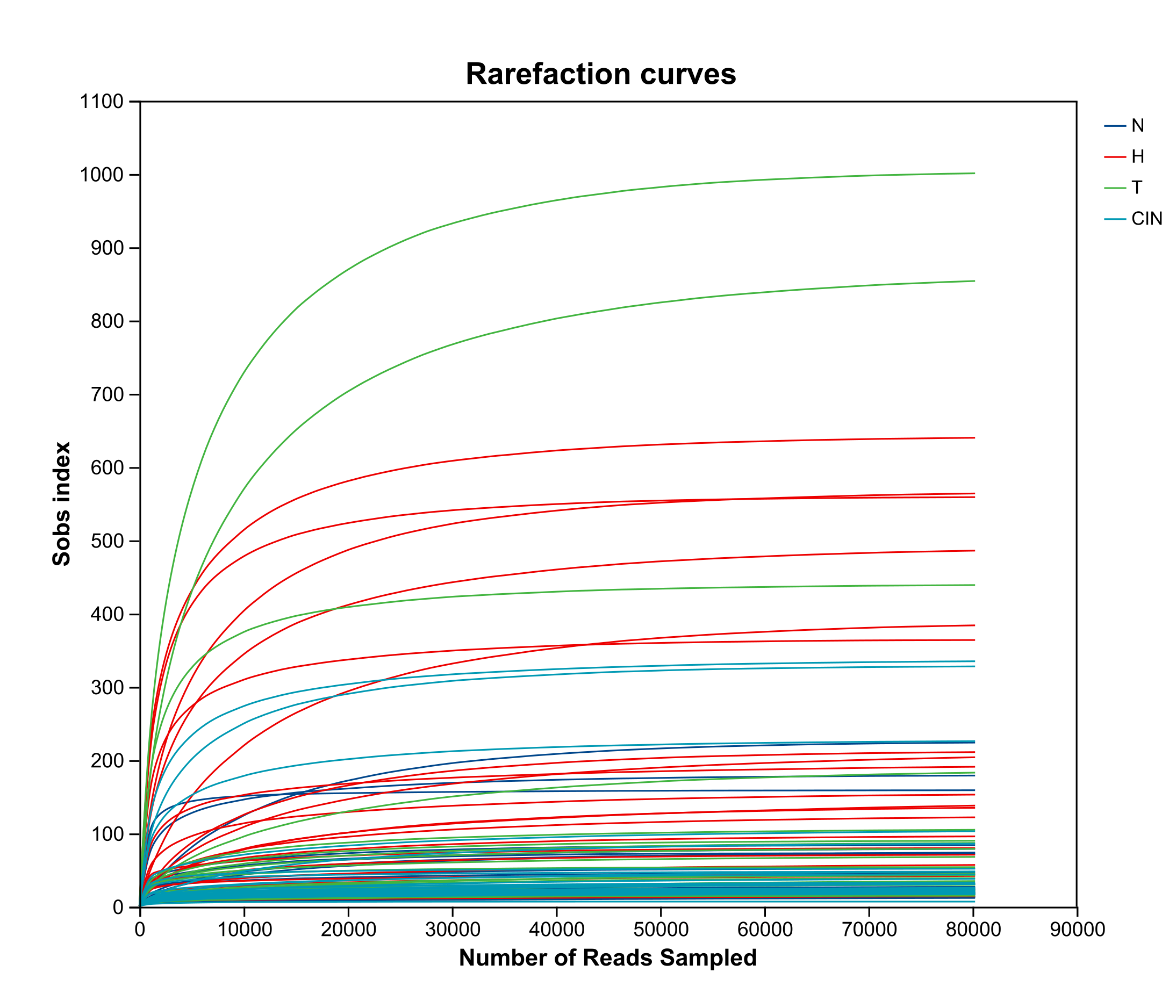

Supplement: S1 Fig — (TIF) [file pone.0343027.s003.tif]

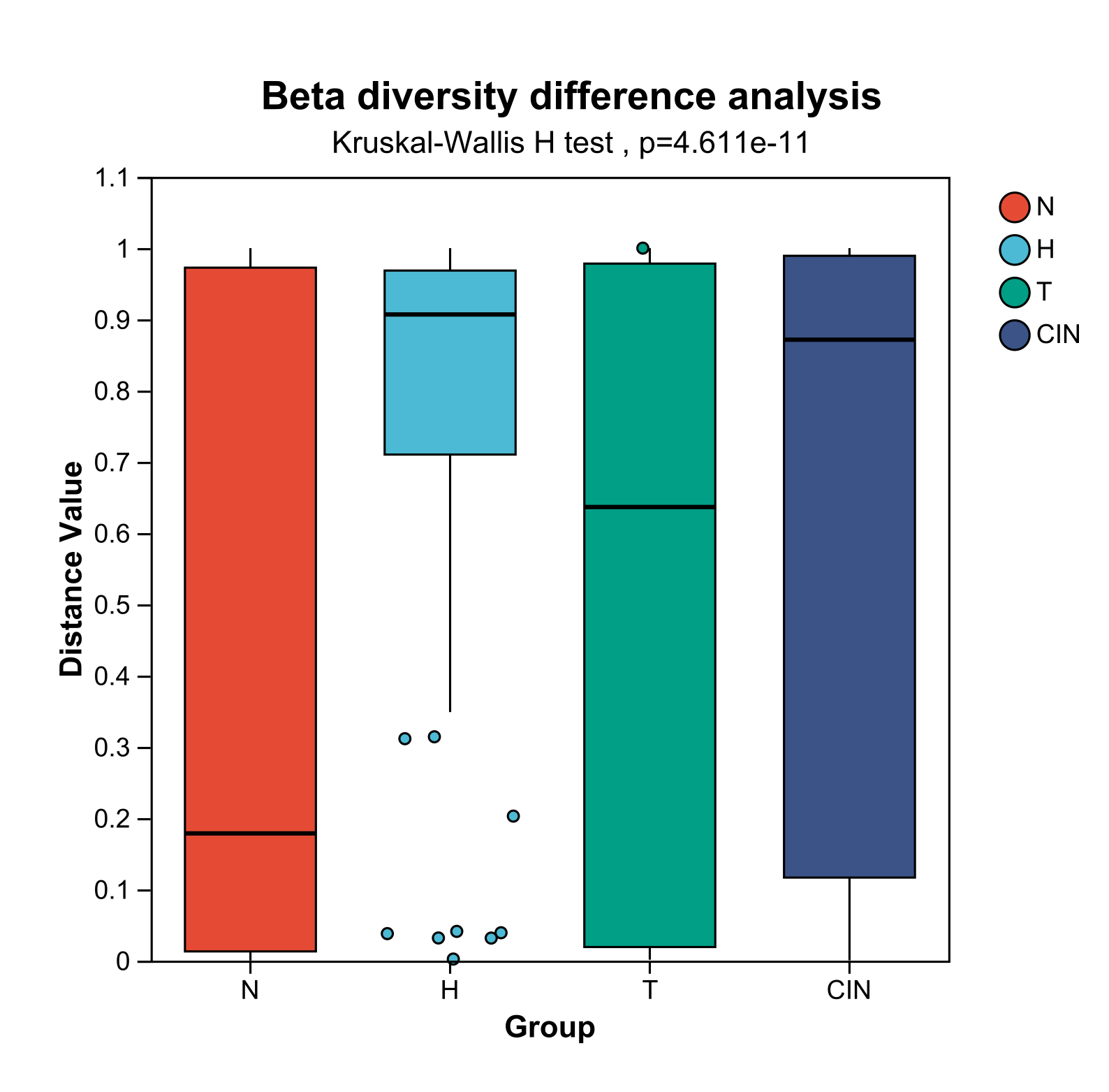

Supplement: S2 Fig — (TIFF) [file pone.0343027.s004.tiff]
